# Extended Follow-up of Local Steroid Injection for Carpal Tunnel Syndrome: A Randomized Clinical Trial

**DOI:** 10.1001/jamanetworkopen.2021.30753

**Published:** 2021-10-22

**Authors:** Manfred Hofer, Jonas Ranstam, Isam Atroshi

**Affiliations:** 1 Department of Orthopedics, Kristianstad Hospital, Kristianstad, Sweden; 2 Department of Clinical Sciences–Orthopedics, Lund University, Lund, Sweden

## Abstract

**Importance:**

Local steroid injection is commonly used in treating patients with idiopathic carpal tunnel syndrome, but evidence regarding long-term efficacy is lacking.

**Objective:**

To assess the long-term treatment effects of local steroid injection for carpal tunnel syndrome.

**Design, Setting, and Participants:**

This exploratory 5-year extended follow-up of a double-blind, placebo-controlled randomized clinical trial was conducted from November 2008 to March 2012 at a university hospital orthopedic department. Participants included patients aged 22 to 69 years with primary idiopathic carpal tunnel syndrome and no prior treatment with local steroid injections. Data were analyzed from May 2018 to August 2018.

**Interventions:**

Patients were randomized to injection of 80 mg methylprednisolone, 40 mg methylprednisolone, or saline.

**Main Outcomes and Measures:**

The coprimary outcomes were the symptom severity score and rate of subsequent carpal tunnel release surgery on the study hand at 5 years. Secondary outcomes were time from injection to surgical treatment, SF-36 bodily pain score, and score on the 11-item disabilities of the arm, shoulder, and hand scale.

**Results:**

A total of 111 participants (mean [SD] age at follow-up, 52.9 [11.6] years; 81 [73.0%] women and 30 [27.0%] men) were randomized, with 37 in the 80 mg methylprednisolone group, 37 in the 40 mg methylprednisolone group, and 37 in the saline placebo group. Complete 5-year follow-up data were obtained from all 111 participants with no dropouts (100% follow-up). At baseline, mean (SD) symptom severity scores were 2.93 (0.85) in the 80 mg methylprednisolone group, 3.13 (0.70) in the 40 mg methylprednisolone group, and 3.18 (0.75) in the placebo group, and at the 5-year follow up, mean (SD) symptom severity scores were 1.51 (0.66) in the 80 mg methylprednisolone group, 1.59 (0.63) in the 40 mg methylprednisolone group, and 1.67 (0.74) in the placebo group. Compared with placebo, there was no significant difference in mean change in symptom severity score from baseline to 5 years for the 80 mg methylprednisolone group (0.14 [95%CI, −0.17 to 0.45]) or the 40 mg methylprednisolone group (0.12 [95%CI, −0.19 to 0.43]). After injection, subsequent surgical treatment on the study hand was performed in 31 participants (83.8%) in the 80 mg methylprednisolone group, 34 participants (91.9%) in the 40 mg methylprednisolone group, and 36 participants (97.3%) in the placebo group; the number of participants who underwent surgical treatment between the 1-year and 5-year follow-ups was 4 participants (10.8%) in the 80 mg methylprednisolone group, 4 participants (10.8%) in the 40 mg methylprednisolone group, and 2 participants (5.4%) in the placebo group. All surgical procedures were conducted while participants and investigators were blinded to type of injection received. The mean (SD) time from injection to surgery was 180 (121) days in the 80 mg methylprednisolone group, 185 (125) days in the 40 mg methylprednisolone group, and 121 (88) days in the placebo group. Kaplan-Meier survival curves showed statistically significant difference in time to surgical treatment (log-rank test: 80 mg methylprednisolone vs placebo, *P* = .002 ; 40 mg methylprednisolone vs placebo, *P* = .02; methylprednisolone 80 mg vs 40 mg, *P* = .37).

**Conclusions and Relevance:**

These findings suggest that in idiopathic carpal tunnel syndrome, local methylprednisolone injection resulted in statistically significant reduction in surgery rates and delay in need for surgery.

**Trial Registration:**

ClinicalTrials.gov Identifiers: NCT00806871 and NCT02652390

## Key Points

**Question:**

Does first-time local steroid injection have long-term treatment effects in carpal tunnel syndrome?

**Findings:**

In 111 adults randomized equally to local injection of 80 mg methylprednisolone, 40 mg methylprednisolone, or placebo, 5-year follow-up showed no differences in symptom severity score improvement among the 3 groups. Participants in the 80 mg methylprednisolone group were less likely to undergo surgical treatment within 5 years than the placebo group.

**Meaning:**

This randomized clinical trial found that local steroid injection resulted in reduction in rate of surgical treatment for individuals with carpal tunnel syndrome but not in significant differences in symptoms at 5 years.

## Teaser

This randomized clinical trial assesses the long-term effects of local steroid injection on symptom severity and rates of subsequent surgery among patients with carpal tunnel syndrome.

## Introduction

Local steroid injection is one of the treatments used in patients with idiopathic carpal tunnel syndrome (CTS). Although the exact mechanism of action of steroid injection for treating CTS is unknown, its short-term efficacy in reducing symptoms has been established. A 2018 systematic review^1^ reported strong evidence that local steroid injection was more effective than placebo at 8 weeks after treatment but that evidence regarding long-term efficacy was lacking. A 2018 randomized clinical trial of CTS in primary care showed short-term advantages of steroid injection compared with wrist splinting.^2^ We conducted a double-blind randomized clinical trial from 2008 to 2012 among 111 participants with idiopathic CTS not previously treated with steroid injection; 37 were allocated to each of 3 groups: 80 mg methylprednisolone, 40 mg methylprednisolone, and placebo.^3^ We found that improvement in the symptom severity score 10 weeks after injection was significantly greater after methylprednisolone than placebo and that the rate of surgery at 1 year was significantly lower in the 80 mg methylprednisolone group than the placebo group (73% vs 92%). To our knowledge, no placebo-controlled randomized clinical trials have reported outcomes of steroid injections beyond 1 year. Although improvement after surgical treatment for CTS has been shown to be durable in the long term,^4^ surgical treatment has substantially higher risk of complications, higher costs, and longer duration of work disability. Another question that has been raised is whether local steroid injection may adversely affect the outcome of subsequent carpal tunnel release surgery.^5^

We have conducted an exploratory 5-year follow-up of our placebo-controlled randomized clinical trial to investigate whether the effect of methylprednisolone injection compared with placebo persists and whether steroid injection is associated with the degree of improvement following subsequent surgical treatment.

## Methods

### Trial Design, Randomization, and Assessments

This double-blind placebo-controlled randomized clinical trial, conducted at 1 university orthopedic department in Sweden between November 2008 and March 2012, has been described previously (Trial Protocol in Supplement 1).^3,6^ The trial and the extended follow-up were approved by the Regional Ethical Review Board, Lund University, Sweden. All participants provided written informed consent. This study follows the Consolidated Standards of Reporting Trials (CONSORT) reporting guideline.

Individuals with idiopathic CTS, according to the Katz diagnostic definition,^7^ who met the trial’s eligibility criteria (eAppendix in Supplement 2) were randomized to 1 of 3 groups: 80 mg methylprednisolone, 40 mg methylprednisolone, and placebo (eFigure in Supplement 2). Based on a computer-generated randomization list (1:1:1 ratio) in varying blocks sequentially numbered, opaque, concealed envelopes containing group assignments were used for randomization by the study nurse. Immediately after randomization, the nurse prepared an injection of methylprednisolone (40 mg/mL) or saline according to the assignment (80 mg methylprednisolone [2 mL], 40 mg methylprednisolone [1 mL + 1 mL saline] or saline [2 mL], each combined with 1 mL lidocaine for a total injected volume of 3 mL) in a covered syringe to mask the surgeon giving the injection and the participant. The surgeon administered the injection into the carpal tunnel using standard technique, and while the needle was withdrawn, a dressing was pressed over the puncture site to conceal the color in case of leakage.^3^

The primary outcomes of the original study were the improvement in symptom severity score at 10 weeks and the rate of surgical treatment at 1 year. At baseline, participants completed the 11-item symptom severity scale, the 2-item Short Form 36 (SF-36) bodily pain scale, and the 11-item disabilities of the arm, shoulder, and hand (QuickDASH) scale. The symptom severity scale measures pain and numbness or tingling and is scored from 1 (no symptoms) to 5 (most severe)^8^; the minimal clinically important difference (MCID) has been estimated at 0.79 points.^9^ The SF-36 bodily pain scale is scored from 0 (worst pain) to 100 (no pain); no CTS-specific MCID values are available, but in individuals with knee osteoarthritis, it has been estimated at approximately 10 points.^10^ The QuickDASH measures difficulties in performing daily activities and is scored from 0 (no disability) to 100 (worst disability)^11,12^; MCID of 6.8 points has been estimated in individuals with nonshoulder upper-extremity conditions (including CTS).^13^ Nerve conduction testing (measurement of median nerve sensory distal latency and median-ulnar sensory latency difference) was performed at baseline and 1 year after injection and classified by a neurophysiologist; sensory latency difference of 1.7 ms or greater, including absent response, was considered severe; 1.0 to 1.6 ms, moderate; 0.6 to 0.9 ms, mild; and less than 0.6 ms, normal.^3^

Of 111 participants aged 22 to 69 years randomized, 37 were assigned to 80 mg methylprednisolone, 37 were assigned to 40 mg methylprednisolone, and 37 were assigned to placebo. All participants received the intervention to which they were assigned. At the initial follow-up evaluations (5 weeks, 10 weeks, 6 months, and 1 year), participants completed the same scales as at baseline and an additional visual analog scale (VAS) about treatment satisfaction, from 0 (very dissatisfied) to 100 (completely satisfied). No repeat injections were given during the trial; participants who did not improve or experienced recurrent symptoms after injection were offered carpal tunnel release surgery. All participants provided complete data for the primary outcomes at 1 year. All participants and investigators remained blinded to group assignment until the last included trial participant had completed the 1-year follow-up, which was March 2012. Thereafter, the randomization codes were broken in April 2012, and in accordance with the trial protocol, participants were informed (through letters mailed in May 2012) about the type of injection they had received. Thus, all participants and investigators were blinded throughout the course of the trial, from November 2008 until April 2012.

### Extended Follow-up

In February 2016, information about this follow-up study, consent forms, and a questionnaire were sent by mail to all 111 trial participants, followed by telephone contact by a research nurse. The questionnaire consisted of the symptom severity sale, QuickDASH scale, SF-36 pain scale, and satisfaction VAS used in the previous evaluations. Participants were also asked about whether they had undergone carpal tunnel release surgery (response choices: “Yes, right hand”; “Yes, left hand”; or “No”). The full medical records of all participants in the trial were examined to ascertain any subsequent surgical treatment or injections.

### Statistical Analysis

The sample size in the trial was calculated before the trial based on the primary outcome of change in symptom severity score from baseline to 10 weeks after injection (coprimary outcome was rate of surgery at 1 year).^3^ We used the χ^2^ test (as prespecified in the protocol for the extended follow-up) to compare the proportion of participants in the 80 mg methylprednisolone group vs the placebo group with regard to the rate of surgical treatment on the study hand during the time from receiving the injection to the 5-year follow-up. We calculated time from injection to surgical treatment among participants who received surgery within 5 years. We compared the rate of and time to surgical treatment in the 3 groups using Kaplan-Meier survival curve. Patient-reported outcomes in each of the methylprednisolone groups were compared with the placebo group using analysis of covariance (ANCOVA) adjusting for baseline factors (sex, age, dominance of the study hand, and the outcome measure’s baseline score). To evaluate whether steroid injection was associated with the outcome of subsequent surgical treatment, we compared the change in symptom severity score in patients who had surgical treatment after methylprednisolone injection with those who underwent surgical treatment after placebo injection using ANCOVA, adjusting for the baseline factors (sex, age, dominance of the study hand, and baseline symptom severity score). Statistical tests were 2-sided, and the level of significance was set at *P* = .05. All statistical tests were conducted with SPSS Statistics version 22 (IBM). Data were analyzed from May 2018 to August 2018.

## Results

Among 111 participants at baseline randomization, the mean (SD) age of the 111 participants was 46.6 (11.6) years, and 81 (73.0%) were women and 30 (27.0%) were men (Table 1). At the 5-year extended follow-up, all 111 trial participants provided complete follow-up data for all outcomes with no dropouts (100% follow-up). Mean (SD) follow-up time was 74.1 (7.2) months, and the mean (SD) age at follow-up was 52.9 (11.6) years. No participant in the placebo group received methylprednisolone, and no participant in the methylprednisolone groups received repeat injections at any time in the trial.

**Table 1.** Participant Characteristics

Characteristic	Mean (SD)		
	Methylprednisolone		Placebo (n = 37)
	80 mg (n = 37)	40 mg (n = 37)	
Baseline (randomization)			
Sex, No. (%)			
Women	26 (70.3)	27 (73.0)	28 (75.7)
Men	11 (29.7)	10 (27.0)	9 (24.3)
Age, y	47 (12)	44 (11)	49 (11)
Dominant hand treated, No. (%)	30 (81.1)	30 (81.1)	28 (75.7)
BMI	26.7 (4.9)	28.9 (7.1)	27.5 (5.5)
Median-ulnar latency difference, ms^a^	1.7 (0.7)	1.4 (0.8)	1.5 (0.8)
1 y after injection	1.2 (0.6)	0.7 (0.5)	0.8 (0.5)
Extended follow-up^b^			
Age, y	53 (12)	50 (11)	55 (11)
Follow-up time, mo	74 (7)	74 (7)	74 (7)
BMI	27.3 (4.2)	27.6 (4.3)	27.7 (5.7)

Abbreviation: BMI, body mass index (calculated as weight in kilograms divided by height in meters squared).

^a^ Absent median nerve sensory response at baseline: 80-mg, 5 patients; 40-mg, 1 patient; placebo, 3 patients.

^b^ Complete follow-up of all participants with no dropouts (sex and dominant hand treated same as at baseline).

### Symptom Severity Score

In all 3 groups, the improvement in the symptom severity score from baseline (before injection) to 5 years was statistically significant. At baseline, mean (SD) symptom severity scores were 2.93 (0.85) in the 80 mg methylprednisolone group, 3.13 (0.70) in the 40 mg methylprednisolone group, and 3.18 (0.75) in the placebo group, and at the 5-year follow-up, mean (SD) symptom severity scores were 1.51 (0.66) in the 80 mg methylprednisolone group, 1.59 (0.63) in the 40 mg methylprednisolone group, and 1.67 (0.74) in the placebo group. There were no statistically significant differences between methylprednisolone groups and placebo (Table 2). The adjusted mean difference in change from baseline between the 80 mg methylprednisolone group and the placebo group was 0.14 (95%CI, −0.17 to 0.45) and between the 40 mg methylprednisolone group and the placebo group was 0.12 (95%CI, −0.19 to 0.43).

**Table 2.** Primary and Secondary Outcomes

Outcome	Mean (SD)		
	Methylprednisolone		Placebo (n = 37)
	80 mg (n = 37)	40 mg (n = 37)	
Symptom severity score^a^			
Baseline	2.93 (0.85)	3.13 (0.70)	3.18 (0.75)
5 y	1.51 (0.66)	1.59 (0.63)	1.67 (0.74)
Adjusted difference, mean (95% CI)^b^	0.14 (−0.17 to 0.45)	0.12 (−0.19 to 0.43)	0 [Reference]
Surgery at 5 y, No. (%)	31 (83.8)	34 (91.9)	36 (97.3)
Time to surgery, d^c^	180 (121)	185 (125)	121 (88)
Difference, mean (95% CI), d	59 (8 to 111)	64 (13 to 116)	0 [Reference]
QuickDASH score^d^			
Baseline	39.9 (22.9)	40.8 (19.2)	44.0 (21.0)
5 y	13.1 (18.2)	16.9 (20.5	19.3 (19.5)
Adjusted difference, mean (95% CI)^b^	4.9 (−3.4 to 13.2)	1.6 (−6.8 to 10.0)	0 [Reference]
SF-36 bodily pain score^e^			
Baseline	45.1 (22.6)	43.5 (20.2)	45.7 (24.0)
5 y	81.4 (24.4)	79.9 (24.2)	78.2 (25.4)
Adjusted difference, mean (95% CI)^b^	−3.7 (−14.9 to 7.6)	−2.8 (−14.3 to 8.6)	0 [Reference]
Treatment satisfaction score^f^			
5 y	77.5 (28.1)	71.4 (26.5)	68.4 (28.3)
Adjusted difference, mean (95% CI)^b^	9.5 (−3.3 to 22.3)	5.2 (−7.8 to 18.2)	0 [Reference]

Abbreviation: QuickDASH, 11-item disabilities of the arm, shoulder, and hand; SF-36, Short Form 36-item.

^a^ Score range: symptom severity scale, 1 (no symptoms) to 5 (most severe).

^b^ Mean differences in change from baseline were adjusted for sex, age, dominance of study hand, and the scale’s baseline score (except for treatment satisfaction); none of the comparisons was statistically significant using analysis of covariance.

^c^ Calculated among the participants who underwent surgical treatment on the study hand during the time between the injection and the 5-year follow-up.

^d^ Range, 0 (no disability) to 100 (most severe).

^e^ Range, 0 (worst) to 100 (best).

^f^ Scored using a visual analog scale. Range, 0 (very dissatisfied) to 100 (completely satisfied).

### Rate of Surgical Treatment

At 5 years after the injection, 101 of 111 participants had undergone carpal tunnel release surgery on the study hand, including 31 participants (83.8%) in the 80 mg methylprednisolone group, 34 participants (91.9%) in the 40 mg methylprednisolone group, and 36 participants (97.3%) in the placebo group (80 mg methylprednisolone vs the placebo, *P* = .05). The number of participants who received surgical treatment between the 1-year and the 5-year follow-ups was 4 participants (10.8%) in the 80 mg methylprednisolone group, 4 participants (10.8%) in the 40 mg methylprednisolone group, and 2 participants (5.4%) in the placebo group. All the participants who received surgical treatment underwent the operation within 16 months of injection, during which time these participants, as well as the investigators and surgeons, were still blinded to group allocation (no participant received surgical treatment after being informed of the type of injection). The mean (SD) time to surgical treatment was 180 (121) days in the 80 mg methylprednisolone group, 185 (125) days in the 40 mg methylprednisolone group, and 121 (88) days in the placebo group (Table 2). The Kaplan-Meier survival curve showed statistically significant difference between methylprednisolone and placebo in rate of and time to surgical treatment (log-rank test: 80 mg methylprednisolone vs placebo, *P* = .002; 40 mg methylprednisolone vs placebo, *P* = .02; 80 mg methylprednisolone vs 40 mg methylprednisolone, *P* = .37) (Figure).

**Figure.**
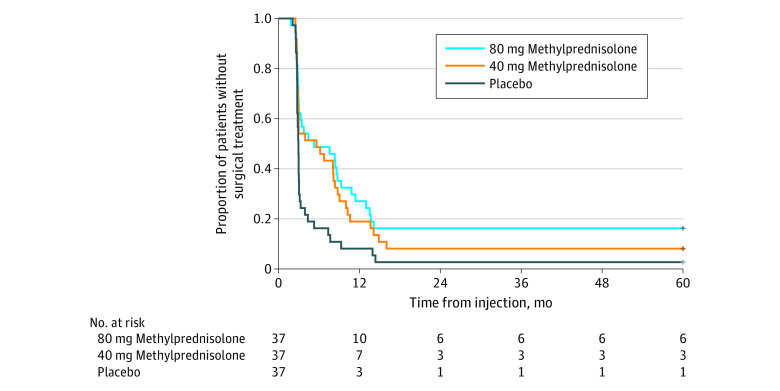
Kaplan-Meier Survival Curves for Time to Surgical Treatment in Participants Who Received 80 mg Methylprednisolone, 40 mg Methylprednisolone, or Placebo Injection

Among participants who received surgical treatment, mean (SD) change in symptom severity score from baseline was −1.44 (0.9) in the 80 mg methylprednisolone group and −1.53 (0.9) in the placebo group; adjusted mean difference was 0.11 (95% CI, −0.23 to 0.46). The mean (SD) change in symptom severity score in patients in the 80 mg methylprednisolone group who did not receive surgery was −1.34 (0.9).

### Other Patient-Reported Outcomes

In all 3 groups, the mean scores for the QuickDASH and SF-36 bodily pain scales improved significantly from baseline to 5 years, and treatment satisfaction was high (Table 2). Mean (SD) QuickDASH scores improved from 39.9 (22.9) at baseline to 13.1 (18.2) at 5 years in the 80 mg methylprednisolone, 40.8 (19.2) at baseline to 16.9 (20.5) in the 40 mg methylprednisolone group, and 44.0 (21.0) at baseline to 19.3 (19.5) at 5 years in the placebo group. Mean (SD) SF-36 scores improved from 45.1 (22.6) at baseline to 81.4 (24.4) at 5 years in the 80 mg methylprednisolone group, 43.5 (20.2) at baseline 79.9 (24.2) at 5 years in the 40 mg methylprednisolone group, and 45.7 (24.0) at baseline to 78.2 (25.4) at 5 years in the placebo group. The mean differences in change from baseline to 5 years in the QuickDASH and SF-36 scores and the mean satisfaction scores at 5 years did not differ significantly between the methylprednisolone and placebo groups (Table 2).

### Adverse Events

Among participants who received surgical treatment, no surgery-related complications were observed. No long-term adverse events related to the trial interventions were reported by the participants or found on medical record review.

## Discussion

This extended follow-up of a double-blind placebo-controlled randomized clinical trial assessing the efficacy of local steroid injection in treating patients with idiopathic CTS found that 90% of the trial participants had undergone surgery within 5 years of injection. The improvement in the symptom severity score, which at 10 weeks after injection was significantly greater in the methylprednisolone groups than in the placebo group, was similar in the 3 groups at this 5-year follow-up, with differences less than the MCID. However, surgical treatment was less likely among participants who had received 80 mg methylprednisolone than among participants who had received placebo (84% vs 97%). In addition, the time from injection to surgical treatment was significantly longer after methylprednisolone injection compared with the placebo group, by approximately 2 months. The participants who did not undergo surgical treatment after methylprednisolone injection had similar symptom severity score improvement at 5 years, indicating maintained efficacy rather than participants choosing not to undergo surgical treatment for other reasons. One area for future research is whether it is possible to identify individuals who are more likely to have durable benefit from steroid injection.

Similar to the symptom severity score, the QuickDASH and SF-36 pain scores showed no statistically significant or clinically relevant differences between the groups, with mean differences being less than the MCID for these scales. Similarly, no statistically significant differences were found in the treatment satisfaction VAS score, and although there is no established MCID for this score, the differences were less than half an SD, a measure that has sometimes been considered to indicate clinically important change.^14^

The long-term benefit of local steroid injection in CTS has been reported in a few nonrandomized studies. A retrospective study of 774 hands treated between 2001 and 2010 and followed with review of medical records until 2014 (median follow-up, 7.4 years) found that 63% received surgical treatment and 32% did not receive subsequent treatment.^15^ Besides that the actual rate of surgical treatment in this type of study is likely to be an underestimate, it also included individuals with rheumatoid arthritis for whom the injection, as expected, was found to be more effective than in idiopathic CTS. In a prospective study that included 113 patients treated with 40 mg methylprednisolone injection, the rate of surgical treatment at 1 year was 67%.^16^ Another prospective study found that among 120 patients with CTS treated with 40 mg methylprednisolone injection (more than 50% received 2-3 repeat injections), only one-third were considered to have had a good outcome at 1 year, mostly those with a good outcome after first injection.^17^

Our results suggest that in the treatment of patients with CTS, a policy of initial treatment with local steroid injection and resorting to surgical treatment in case of inadequate symptom improvement or recurrence may delay and to some extent reduce the rate of surgical treatment in the long term. Our study shows important improvement in quality of life, as shown by the large improvement in the SF-36 bodily pain score, from approximately 45 before treatment to approximately 80 at this long-term follow-up. In fact, this improvement in the SF-36 bodily pain score is similar in magnitude to that reported in 151 patients with osteoarthritis before and 7 years after total hip arthroplasty.^18^ This is in addition to the large improvement in patient-reported hand symptoms and activity limitations and improvement in median nerve impairment already shown 1 year after treatment. However, in most participants, these effects were largely found in surgical treatment or improvement over time.

Our study also found that symptom improvement in patients who underwent carpal tunnel release surgery after steroid injection did not differ from that in participants who underwent surgical treatment after placebo injection. Our results do not address whether repeated steroid injections may adversely affect outcomes of subsequent surgical treatment.

Aside from the complete follow-up, this randomized clinical trial is the only double-blind placebo-controlled trial assessing the efficacy of local steroid injection in CTS beyond 1 year, to our knowledge. The results are generalizable to individuals with idiopathic CTS, no severe sensory loss or muscle atrophy, and no previous treatment with local steroid injection

### Limitations

This study has some limitations. The large proportion of participants who proceeded to carpal tunnel release because of persistent or recurrent symptoms after steroid injection meant that the difference in rate of surgical treatment was small. One limitation is that the trial was double-blind only up to the time when the last trial participant had completed the 1-year follow-up, after which all participants were informed about the type of injection they had received, in accordance with the trial protocol. However, all carpal tunnel release surgical procedures after injection were performed while all participants, investigators, and operating surgeons were still blinded to group allocation; therefore, this factor did not affect the rate of and the time to surgery. Participants were no longer blinded when they responded to the questionnaire at the long-term follow-up, which could have influenced the scores for the patient-reported measures; however there were no statistically significant or clinically relevant differences between the groups in any of the scores. Other limitations include a single center and the moderate sample size. A possible limitation is that with the injection technique used, a common technique in clinical practice, the exact location of the injection in the carpal tunnel may not have been identical in all injections. However, all injections were given by a single specialist blinded to the type of injection, and the significant improvement in symptom severity score at 10 weeks compared with the placebo injection shows the efficacy of the steroid injected with this technique. It is currently unknown whether ultrasonography-guided steroid injections could provide a more durable benefit.

## Conclusions

This randomized clinical trial found that in patients with idiopathic carpal tunnel syndrome, local methylprednisolone injection resulted in significant delay in need for surgical treatment but only a small reduction in rate of surgery. Symptoms and activity limitations related to carpal tunnel syndrome had improved significantly 5 years after injection for intervention and placebo groups, and the improvements were similar among participants who received subsequent surgical treatment and those who did not.

## Notes

Supplement 1.
Trial Protocol

Supplement 2. **eAppendix.** Trial Eligibility Criteria **eFigure.** Trial Flowchart

Supplement 3.
Data Sharing Statement
