# Supplementary material for: Extended Follow-up of Local Steroid Injection for Carpal Tunnel Syndrome: A Randomized Clinical Trial
Source: JAMA Netw Open. 2021 Oct 22;4(10):e2130753. doi: 10.1001/jamanetworkopen.2021.30753 (PMC8536954; doi:10.1001/jamanetworkopen.2021.30753)
Supplement: Supplement 1. — Trial Protocol [file jamanetwopen-e2130753-s001.pdf]

Supplement 1

Contents:

1. Pre-enrollment Trial Protocol

2. Protocol for the Extended Follow-up

3. Ethical Review Attachments for the Extended Follow-up

1. Pre-enrollment Trial Protocol

SYNOPSIS
6. CLINICAL TRIAL PROTOCOL AND PROTOCOL AMENDMENT(S)
However, sitespecific information may be provided on separate protocol page(s), or addressed in a separate agreement, and some of the information listed below may be contained in other protocol referenced documents, such as an Investigator´s Brochure.

6.1 General Information

6.1.1 Protocol title, protocol identifying number, and date.
Local steroid injection in the treatment of idiopathic carpal tunnel syndrome: A randomized double-blind placebo-controlled trial

Version 2
Dated 2008-07-18

6.1.2 Name and address of the sponsor and monitor (if other than the sponsor).
Magnus Flondell (Sponsor)
Ingela Ranebo (monitor)
Region Skåne
Sjukhusorganisationen Hässleholm
Esplanadgatan
281 25 Hässleholm

6.1.3 Name and title of the person(s) authorized to sign the protocol and the protocol amendment(s) for the sponsor.
Isam Atroshi MD, PhD,Associate professor
Magnus Flondell MD

6.1.4 Name, title, address, and telephone number(s) of the sponsor's medical expert (or dentist when appropriate) for the trial.
Isam Atroshi MD, PhD, Associate professor 044-3091279
Magnus Flondell MD 0451-296418
Region Skåne
Sjukhusorganisationen Hässleholm
Esplanadgatan
281 25 Hässleholm

6.1.5 Name and title of the investigator(s) who is (are) responsible for conducting the trial, and the address and telephone number(s) of the trial site(s).
Isam Atroshi
Magnus Flondell
Region Skåne
Sjukhusorganisationen Hässleholm
Esplanadgatan
281 25 Hässleholm
0451-296000, 044-3091000
6.1.6 Name, title, address, and telephone number(s) of the qualified physician (or dentist, if applicable), who is responsible for all trial-site related medical (or dental) decisions (if other than investigator).

Investigator
6.1.7 Name(s) and address(es) of the clinical laboratory(ies) and other medical and/or technical department(s) and/or institutions involved in the trial.

Pia Gunnarsson, reg nurse
Responsible for nerve conduction measurement
Ortopediska kliniken
Centralsjukhuset I Kristianstad

Manfred Hofer
Physiotherapist with experience of clinical hand studies
Rehabiliterings kliniken
Centralsjuknuset Kristianstad

6.2 Background Information
6.2.1 Name and description of the investigational product(s).
Depomedrol 40mg/ml utan konserveringsmedel
Xylocain 10mg/ml
Natriumklorid 0.9%

6.2.2 A summary of findings from nonclinical studies that potentially have clinical significance and from clinical trials that are relevant to the trial.
Carpal tunnel syndrome (CTS) is the most common peripheral compression neuropathy in the upper extremity with a prevalence of 3.7% in the general population.[1] CTS of mild severity is commonly treated with wrist splint but surgical treatment is often required.[2] Carpal tunnel release is one of the most common surgical procedures with an annual incidence of 120-140 /100000 per year.[3] Thus the number of carpal tunnel release operations performed annually in Sweden may be up to an estimated 13000. The prevalence of persons in the general population who have moderate or severe CTS but have not sought care or been diagnosed correctly is almost 1%.[4]

Although carpal tunnel release has been shown to produce good outcomes regarding relief of symptom caused by CTS,[5] it has several disadvantages including surgery-related pain and hand weakness.[6] These are common problems and may last several months after surgery. Other less common complications include wound infection, chronic regional pain syndrome, and nerve injuries.[7,8] In addition, surgery is associated with direct costs as well as indirect costs related to work absence after surgery. The majority of patients who undergo CTS surgery require sick leave for a varying length of time depending on the degree of postoperative morbidity and the type of work.[9] The median time of work absence has been 4 weeks in several studies, with a proportion of patients having long-term work disability.[9] The economic impact of being out of work due to CTS surgery can be substantial both for the patients and the society. Consequently avoiding sick leave would be an important advantage for non-operative treatment.

Although many alternatives to surgery have been proposed there is little evidence to support the efficacy of most of these treatments.[10] Steroid injection into or proximal to the carpal tunnel is widely practiced as a non-operative treatment for patients with

idiopathic CTS particularly in the United States.[11] However the evidence for its efficacy has not been established.

In a recent Cochrane review of local corticosteroid injection for CTS, the authors concluded that there was evidence supporting clinical improvement at one month compared to placebo but no significant improvement after 8 weeks compared to non-steroidal anti-inflammatory drugs or wrist splint.[12] None of the studies contained a double blinded comparison with a follow-up of more than one month.
In a study that compared surgery with steroid injections, the authors reported similar effects in the short term but outcomes were not measured with standardized validated instruments.[13] Previous studies comparing steroid injection with surgery have not specifically involved patients whose CTS was of such severity that surgery was clearly indicated. Obviously, if the study population includes patients with less severe CTS the comparison may be biased. A self-administered outcome instrument for the assessment of symptoms, function and patient satisfaction in CTS has been shown to be reliable, valid and sensitive to clinical change.[14,15] Because the main effects of treatment are symptoms and improvement in hand function it is important that these outcomes be measured with reliable and valid instruments
Outcome measures
The CTS symptoms severity scale is a validated questionnaire inquiring about severity, frequency and duration of night and daytime pain and numbness or tingling.[12,13] The CTS score ranges from 1 (no symptoms) to 6 (most severe symptoms). The *Quick*DASH is 11-item disabilities of the arm shoulder and hand (DASH) questionnaire that inquires about difficulties in performing daily activities, yielding a score from 0 (no disability) to 100 (worst possible disability).[15] The SF-6D is a validated health utility measure that is used to compare cost-effectiveness of different treatments and the value ranges from -0.11 (worst health) to 1.0 (perfect health).[16] Adverse events will be recorded.

At baseline and follow-up a physical examinations will be performed by the same physiotherapist with many years experience in hand therapy. Semmes-Weinstein monofilament and two-point discrimination tests of sensation will be performed on the radial and ulnar aspects of each finger; two-point discrimination testing will be started with a distance of 4 mm and successively increased by 2 mm until the correct tactile discrimination is recorded. Grip strength and 3-point pinch strength, measured with the Baseline dynamometer and pinch gauge (Chattanooga Group, Hixson, Tennessee, USA), respectively, will be recorded (three trials for each hand). Before the 52-week examination, the patients will be instructed not to discuss their treatment with the assessor and will have their palm covered with a dressing concealing possible scar after carpal tunnel release. The assessor will thus be blinded to whether the patient had or had not undergone surgery after the injection. After examination the patient will proceed to nerve conduction testing keeping the same palm dressing so that the examiner also will be blinded.

6.2.3 Summary of the known and potential risks and benefits, if any, to human subjects.
***Risks*: For potential side effects see resumé for the drugs used in the study.**
Intra-neural injection with nerve damage (very few cases reported in the literature)
Infection from the puncture site
***Benefits*: Anti-inflammatory effect curing CTS**

6.2.4 Description of and justification for the route of administration, dosage, dosage

regimen, and treatment period(s).
The choice of study drug, doses and placebo is based on current treatment routines, the Swedish Pharmacopee (FASS), and the cited literature.
Treatment with injection in the carpal canal has been tried in many studies in the dosage proposed 40mg Methylprednisolone. It is currently used in that manner in clinical use in patients with rheumatoid arthritis with CTS. The effect in earlier studies has been inadequate, which may partly be related to inadequate dose. Treatment of tenosynovitis-related swelling may more likely be effective with 80mg Methylprednisolone. The recommendation for injections in the carpal canal is 40 to 80mg and we hypothesize that 80 mg would be more effective than 40 mg in relieving symptoms of carpal tunnel syndrome.

6.2.5 A statement that the trial will be conducted in compliance with the protocol, GCP and the applicable regulatory requirement(s).
The trial will be conducted in compliance with the protocol, GCP and the applicable regulatory requirement(s).

6.2.6 Description of the population to be studied.
Patients referred by primary care physicians to the Department of Orthopedics Hässleholm-Kristianstad because of suspected carpal tunnel syndrome are examined by orthopedic surgeons at the outpatient clinic and enrolled according to prespecified inclusion and exclusion criteria.

6.2.7 References to literature and data that are relevant to the trial, and that provide background for the trial.

References

1. Atroshi I, Gummesson C, Johnsson R, Ornstein E, Ranstam J, Rosen I. Prevalence of carpal tunnel syndrome in a general population. JAMA 1999;282:153-158.
2. Gerritsen AA, De Vet HC, Scholten RJ, Van Tulder MW, Bouter LM. Enabling meta-analysis in systematic reviews on carpal tunnel syndrome. J Hand Surg [Am] 2002;27:828-832.
3. Atroshi I. Carpal tunnel syndrome: prevalence, electrodiagnosis and outcome instruments. 1999. Lund University, Lund, Sweden.
4. Atroshi I, Gummesson C, Johnsson R, McCabe SJ, Ornstein E. Severe carpal tunnel syndrome potentially needing surgical treatment in a general population. J Hand Surg [Am] 2003;28:639-644.
5. Atroshi I, Gummesson C, Johnsson R, Sprinchorn A. Symptoms, disability, and quality of life in patients with carpal tunnel syndrome. J Hand Surg 1999;24A:398-404.
6. Wintman BI, Winters SC, Gelberman RH, Katz JN. Carpal tunnel release. Correlations with preoperative symptomatology [see comments]. Clin Orthop 1996;135-145.
7. Ahlberg J, Johansson H, Widenfalk B. [Disabling injuries following carpal tunnel syndrome surgery]. Lakartidningen 2007;104:2884-2886.
8. Benson LS, Bare AA, Nagle DJ, Harder VS, Williams CS, Visotsky JL. Complications of endoscopic and open carpal tunnel release. Arthroscopy 2006;22:919-24, 924.
9. Atroshi I, Larsson GU, Ornstein E, Hofer M, Johnsson R, Ranstam J. Outcomes of endoscopic surgery compared with open surgery for carpal tunnel syndrome among employed patients: randomised controlled trial. BMJ 2006;332:1473-1476.
10. O'Connor D, Marshall S, Massy-Westropp N. Non-surgical treatment (other than steroid injection) for carpal tunnel syndrome. Cochrane Database Syst Rev 2003;CD003219
11. American Society for Surgery of the Hand. Carpal tunnel syndrome. Available from: http://www.assh.org/Content/NavigationMenu/PatientsPublic/HandConditions/CarpalTunnelSyndrome/carpal_tunnel.pdf. Accessed 1-1-0080.
12. Marshall S, Tardif G, Ashworth N. Local corticosteroid injection for carpal tunnel syndrome. Cochrane Database Syst Rev 2007;CD001554

13. Ly-Pen D, Andreu JL, de BG, Sanchez-Olaso A, Millan I. Surgical decompression versus local steroid injection in carpal tunnel syndrome: a one-year, prospective, randomized, open, controlled clinical trial. Arthritis Rheum 2005;52:612-619.
14. Levine DW, Simmons BP, Koris MJ, Daltroy LH, Hohl GG, Fossel AH, Katz JN. A self-administered questionnaire for the assessment of severity of symptoms and functional status in carpal tunnel syndrome. J Bone Joint Surg 1993;75A:1585-1592.
15. Atroshi I, Johnsson R, Sprinchorn A. Self-administered outcome instrument in carpal tunnel syndrome: reliability, validity and responsiveness evaluated in 102 patients. Acta Orthop Scand 1998;69:82-88.
16. Gummesson C, Ward MM, Atroshi I. The shortened disabilities of the arm, shoulder and hand questionnaire (QuickDASH): validity and reliability based on responses within the full-length DASH. BMC Musculoskelet Disord 2006;7:44
17. Atroshi I, Gummesson C, McCabe SJ, Ornstein E. The SF-6D health utility index in carpal tunnel syndrome. J Hand Surg Eur Vol 2007;32:198-202.
18. Liang MH, Fossel AH, Larson MG. Comparisons of five health status instruments for orthopedic evaluation. Med Care 1990;28:632-642.

© EMEA 2006 37

6.3 Trial Objectives and Purpose
A detailed description of the objectives and the purpose of the trial.

TRIAL OBJECTIVES
To assess the efficacy of injecting steroid into the carpal tunnel in relieving symptoms of carpal tunnel syndrome for at least 1 year with outcomes measured with valid standardized outcome instruments.

6.4 Trial Design
The scientific integrity of the trial and the credibility of the data from the trial depend substantially on the trial design. A description of the trial design, should include:

6.4.1 A specific statement of the primary endpoints and the secondary endpoints, if any, to be measured during the trial.
Primary endpoint

1.-The score change on the validated CTS symptom severity scale at 10 weeks after treatment.
Comparing:
1. 80 mg Depomedrol vs placebo
2. 40 mg Depomedrol vs placebo
3. 80 mg Depomedrol vs 40 mg Depomedrol

2.-Rate of surgery in rank order 52,24,12 weeks after treatment
Comparing:
1. 80 mg Depomedrol vs placebo
2. 40 mg Depomedrol vs placebo
3. 80 mg Depomedrol vs 40 mg Depomedrol

Secondary endpoints

1. The score change on the validated CTS symptom severity scale at 52 weeks after treatment.
2. Time to surgery
3. QuickDASH score at 10 and 52 weeks
4. SF-6D score at 10 and 52 weeks
5. Patient satisfaction with treatment at 10 and 52 weeks

6.4.2 A description of the type/design of trial to be conducted (e.g. double-blind, placebocontrolled, parallel design) and a schematic diagram of trial design, procedures and stages.
The trial is randomized prospective double-blind placebo controlled dose-response clinical trial.
Patients referred by primary care physicians to the Department of Orthopedics Hässleholm-Kristianstad are examined by orthopedic surgeons at the outpatient clinic. Those with a clinical diagnosis of CTS who have tried wrist splint and whose symptom severity is judged by the examining surgeon to warrant surgery are offered to be put on the waiting list for carpal tunnel release. Patients who accept this are put on the waiting list, which means a waiting time of approximately 3 months. A trial investigator contacts eligible patients and explains for them the aims and advantages and disadvantages of the trial and asks them if they wanted to participate. Patients who give informed consent

will be asked to attend the outpatient clinic for a physical examination followed by allocation to one of the three trial groups. The recruited patients are assigned to a treatment group at the outpatient clinic according to a randomization list based on the patients sequence number of entry and uniformly distributed random numbers. Immediately following the allocation the patients will receive the assigned treatment. Recruitment to the trial according to the plan described above is believed to guarantee a representative sample from the patient population residing in the catchment area. All patients are asked for informed consent before any study procedure is performed. The patients will be recorded in a screening/inclusion/randomization list.

TRIAL FLOW SHEET:

	Pre-screen	Visit 1 Baseline	Visit 2 5 w +5 d	Visit 3 10 wk ±7 d	*Surgery* 12 wk ±7 d	Visit 4 24 wk ±7 d	Visit 5 52 wk ±7 d
Diagnosis/inclusion /exclusion criteria	X						
Demographics		X					
Randomization to Treatment		X					
Physical examination<br>Two-point discrimination<br>Monofilament test<br>Grip strength<br>Pinch strength		X		X		X	X
Nerve conduction testing		X					X
Outcome questionnaires		X	X	X		X	X
Rate of surgery					X	X	X
Sick-leave report		X	X	X		X	X
Adverse events		X	X	X		X	
Study medication		X					
Concomitant medication		X	X	X		X	

6.4.3 A description of the measures taken to minimize/avoid bias, including:
a)Randomization.

A randomization list of 90 items will be generated by computer. Based on the list sequentially numbered sealed opaque envelopes containing cards with group assignments will be prepared. These sealed envelopes marked with the patients' sequential numbers are kept at the Orthopedic Department's Outpatient clinic. When a patient is enrolled and written informed consent obtained, the nurse will open the envelope with the lowest number and prepare the injection according to the card. She will then sign date, name and sequential number on the medication list. The nurse preparing the drug is responsible for putting the card in a new envelope marked with the sequential number. This card will be kept in a box with the study medication. As backup, a sealed envelope with the randomization database is kept together with the study drugs, in the locked area reserved for drugs at the outpatient department. In case

the blinding needs to be broken for a patient, the nurse will open the envelope and extract the information for the patient. The envelope will be signed with name and date, and given to a monitor.
b)Blinding
The nurse at the Outpatient clinic will prepare the medication with a label covering the syringe. The injection will then be given by the examiner. Placebo is clear and Depomedrol is a white suspension. In subfascial injections such as in the carpal canal leakage from the injection canal is rare. The risk of revealing what is administered is small. Also the examiner who will discuss with the patient and decide whether the patient will proceed with operation or only continue follow-up will be a different person than the treating surgeon and will be blinded to the treatment assignment. The follow-up at weeks 24 and 52 will be done by a physiotherapist blinded to the treatment assignment.

.
6.4.4 A description of the trial treatment(s) and the dosage and dosage regimen of the investigational product(s). Also include a description of the dosage form, packaging, and labelling of the investigational product(s).
TRIAL TREATMENTS

Group A : 1 ml 40 mg Methylprednisolone + 1 ml 10mg Lidocaine + 1 ml saline

Group B: 2 ml 80mg Methylprednisolone + 1 ml 10mg Lidocaine

Group C: 1 ml 10 mg Lidocaine + 2 ml saline

The vials and packaging will be the original delivered from Pfizer (Depomedrol), AstraZeneca (Xylocain) and Bayer (saline)

The drugs will be labelled with the name of the sponsor, name, telephone and address of the investigator, name of the investigation and batch, and refer to dosage instructions in the protocol, and marked "for use in clinical trial". A sample of the labelling is enclosed with the application.
6.4.5 The expected duration of subject participation, and a description of the sequence and duration of all trial periods, including follow-up, if any.
Patients referred by primary care physicians to the Department of Orthopedics Hässleholm-Kristianstad are examined by orthopedic surgeons at the Outpatient clinic. Patients with a clinical diagnosis of CTS who have tried wrist splint and whose symptom severity is judged by the examining surgeon to warrant surgery are offered to be put on the waiting list for carpal tunnel release. Patients who accept this are put on the waiting list, which means a waiting time of approximately 3 months. A trial investigator contacts eligible patients and explains for them the aims and advantages and disadvantages of the trial and asks them if they wanted to participate. Patients who agree will be asked to attend the outpatient clinic for a physical examination followed by allocation to one of the three trial groups. The recruited patients are assigned to a treatment group at the outpatient clinic according to a randomization list based on the patients sequence number of entry and uniformly distributed random numbers. Immediately following the allocation the patients will receive the assigned treatment.
<u>At Baseline visit</u>: demographics, earlier and ongoing disease, concomitant medication will be recorded. Physical examination will be performed. Patients will complete a

questionnaire that includes the CTS symptoms severity scale, the QuickDASH, the SF-6D and demographic data.
<u>At 5 weeks after treatment, telephone contact</u>: the patients will be asked to complete the CTS symptoms severity scale and AE and concomitant medication will be recorded.
<u>At 10 weeks after treatment, visit:</u> the patients will be examined by a physiotherapist blinded to the patient's group assignment and the patient will be asked whether she/he wants to proceed with surgery as planned at 12 weeks after treatment or whether the patient had experienced adequate symptom relief that she/he wants to continue follow-up and not have the planned surgery at this stage. A similar evaluation to baseline is made and the portion with same indications for surgery will be recorded. All patients will complete the CTS symptoms severity scale. AE, and concomitant medication will be recorded.
<u>At 12 weeks</u>: patients who decided to have surgery will be operated on as scheduled at 12 weeks.
<u>At 24 weeks after treatment, visit</u>: all patients will be examined, asked to complete a questionnaire including the CTS symptoms severity scale, the QuickDASH, the SF-6D as well as rate their satisfaction with the treatment on a visual analog scale. AE and concomitant medication will be recorded.
<u>At 52 weeks after treatment, visit</u>: all patients will be asked to attend a physical examination and nerve conduction tests and complete the questionnaires.

6.4.6 A description of the "stopping rules" or "discontinuation criteria" for individual subjects, parts of trial and entire trial.

6.4.7 Accountability procedures for the investigational product(s), including the placebo(s) and comparator(s), if any.
The investigators will discuss on a regular basis the progress of the trial. The investigators will have the right to terminate the trial at any time in case of serious adverse events or if special circumstances should occur, making further patient treatment impossible. Interim analysis will be performed by fellow researcher not involved in study after completion of half of the patients if the adverse events are not in accordance to the product resumes.
6.4.8 Maintenance of trial treatment randomization codes and procedures for breaking codes.
The sealed envelopes marked with the patients' sequential numbers are kept at the Orthopedic Department's Outpatient clinic. When a patient is enrolled and written informed consent obtained the nurse will open the envelope with the lowest number and prepare the injection according to the card. She will then sign date, name and sequential number on the medication list. The nurse administering the drug is responsible for putting the card in a new envelope marked with sequential number. This card will be kept in a box with the study medication. As backup, a sealed envelope with the randomization database is kept together with the study drugs, in the locked area reserved for drugs at the Outpatients department.
6.4.9 The identification of any data to be recorded directly on the CRFs (i.e. no prior written or electronic record of data), and to be considered to be source data.
In case the blinding needs to be broken for a patient, the nurse will open the envelope and extract the information for the patient. The envelope will be signed with name and date, and given to a monitor.

6.5 Selection and Withdrawal of Subjects

6.5.1 Subject inclusion criteria.

Inclusion Criteria

- **Primary, idiopathic CTS**
- **Age 18-70 years, either gender**
- **Symptom duration of at least 3 months and inadequate response to wrist splint**
- **Symptoms of classic or probable CTS according to the diagnostic criteria in Katz hand diagram**
- **Nerve conduction studies showing median neuropathy at the wrist and no other abnormalities or, in the absence of abnormal nerve conduction study results, 2 surgeons should independently diagnose the patient with CTS.**

6.5.2 Subject exclusion criteria.

Exclusion Criteria

- **Previous steroid injection for CTS in the same wrist**
- **Inflammatory joint disease, diabetes mellitus**
- **Vibration-induced neuropathy, polyneuropathy**
- **Pregnancy**
- **Trauma to the affected hand in the previous year**
- **Previous CTS surgery in the affected hand**
- **Inability to complete questionnaires due to language problem or cognitive disorder**
- **Severe medical illness**
- **Known abuse of drugs and/or alcohol**

6.5.3 Subject withdrawal criteria (i.e. terminating investigational product treatment/trial treatment) and procedures specifying:
a) When and how to withdraw subjects from the trial/ investigational product treatment.
b) The type and timing of the data to be collected for withdrawn subjects.

c) Whether and how subjects are to be replaced.
d) The follow-up for subjects withdrawn from investigational product treatment/trial treatment.

Trial termination

Patients leaving the trial before the trial endpoint, 52 weeks, should, if possible, go through the same final evaluations as patients completing the trial according to the protocol.
Every reasonable effort should be made to maintain patient protocol compliance and participation in the trial. Should a patient withdraw or be prematurely terminated from the trial for any reason, the patient will be strongly encouraged to return for a final visit.

Withdrawals

Patients are free to withdraw from the trial at any time without the need to give reasons, and without prejudice to further treatment. Patients may be withdrawn from the trial at any time at the discretion of the investigator. The reason for this should be documented. The total number of the study will be increased in accordance with the number of patients not completing the study (in order to maintain the power of the study).
The drugs are injected into the tissue only once and have no antidote so withdrawal from treatment is not a relevant issue.

6.6 Treatment of Subjects
6.6.1 The treatment(s) to be administered, including the name(s) of all the product(s), the dose(s), the dosing schedule(s), the route/mode(s) of administration, and the treatment period(s), including the follow-up period(s) for subjects for each investigational product treatment/trial treatment group/arm of the trial.
Group A : 1 ml 40 mg Methylprednisolone (Depomedrol 40mg/ml) + 1 ml 10mg Lidocaine (Xylocain 10mg/ml) + 1 ml saline (NaCl Bayer)

Group B: 2 ml 80mg Methylprednisolone (Depomedrol 40mg/ml) + 1 ml 10mg Lidocaine(Xylocain 10mg/ml)

Group C: 1 ml 10 mg Lidocaine (Xylocain 10mg/ml) + 2 ml saline (NaCl Bayer)

The mixture of drugs is prepared by the nurse in the outpatients department of orthopedics. This m.o. is standard procedure for Depomedrol injection and all nurses have experience in performing this task. The drugs will be injected subfascially in the soft tissue of the carpal canal once by the investigator at baseline. All patients included in the study will receive the same follow-up, AE follow-up is 26 w. Application for exception on handling the drug is included with the application to the competent authority

6.6.2 Medication(s)/treatment(s) permitted (including rescue medication) and not permitted before and/or during the trial.
Ciklosporin
Ketokonazol
Itrakonazol

Se dose adjustments in the product resumes

6.6.3 Procedures for monitoring subject compliance.
Not applicable since there is only one injection given by the investigator
6.7 Assessment of Efficacy
6.7.1 Specification of the efficacy parameters.
Primary endpoint

1.-The CTS symptom severity score at 10 weeks after treatment.
Comparing:
1. **80 mg Depomedrol vs placebo**
2. **40 mg Depomedrol vs placebo**
3. **80 mg Depomedrol vs 40 mg Depomedrol**

The study design does not allow a direct comparison between injection and surgery

2.-Rate of surgery in rank order 52,24,12 weeks
Comparing:
1. **80 mg Depomedrol vs placebo**
2. **40 mg Depomedrol vs placebo**
3. **80 mg Depomedrol vs 40 mg Depomedrol**

Secondary endpoints

1. The CTS symptom severity score at 52 weeks after treatment.
2. Time to surgery
3. QuickDASH score
4. SF-6D score
5. Patient satisfaction with the results of treatment (VAS scale)

6.7.2 Methods and timing for assessing, recording, and analysing of efficacy parameters.
Outcome Measures

The CTS symptom severity scale is a validated self-administered 11-item questionnaire inquiring about severity, frequency and duration of symptoms experienced by the patient in the past two weeks including nocturnal and daytime pain and numbness or tingling. Each item has 5 response options ranging from 1 (no symptom) to 5 (most severe symptom). The symptom score is the mean of all answered items with higher scores indicating worse symptoms. The *Quick*DASH is a short form of the disabilities of the arm shoulder and hand (DASH) questionnaire, a widely used outcome measure for upper extremity disorders including CTS.[16] The *Quick*DASH consists of 11 items concerning difficulties in performing activities of daily living with 5 response options (no difficulty to unable to perform). The SF-6D is a validated health utility measure derived from 11 items of the SF-36 health status and quality of life questionnaire; the SF-6D can be used to compare cost-effectiveness of different treatments.[17]

6.8 Assessment of Safety

6.8.1 Specification of safety parameters.
Definition of adverse events directly related to injection

- **AE**
  - ◊ **Nerve injury normalized within 10 weeks**
  - ◊ **Infection resolved without operative treatment**

- **SAE**
  - ◊ **Nerve injury not normalized within 10 weeks**
  - ◊ **infection requiring operative treatment**

General adverse events in accordance to ICH-GCP
AE
All diseases and symptoms not present at baseline will be recorded. If exacerbation of symptoms or concomitant disease occurs it will be recorded
SAE
- **Death**
- **Life-threatening disease**
- **Disease or symptom warranting hospitalization or prolongation of hospitalization with more than 24 hours.**
- **Disease or symptom causing a permanent disability**
- **Congenital malformation/defect**

Other important medical event

SUSAR = "Suspected, Unexpected Serious Adverse Reaction will be recorded and forwarded to the responsible authority within 7 days if serious and 15 days if not

6.8.2 The methods and timing for assessing, recording, and analysing safety parameters.

Safety parameters will be gathered at baseline, 5, 10, 24 weeks and analysis of AE and SAE will be done by comparing rates to the rates recorded in the product resumes. The statistician will be involved in evaluating the rates during and after the study

6.8.3 Procedures for eliciting reports of and for recording and reporting adverse event and intercurrent illnesses.

The nature and severity of adverse events will be recorded in CRF. All regarded serious will be reported to the proper authority within 1 week

6.8.4 The type and duration of the follow-up of subjects after adverse events.

Adverse events will be recorded at baseline, 5, 10, 24 weeks and if the patient contacts the unit with a symptom. Follow-up of suspected or confirmed infection or intra-neural injection at puncture site will be according to clinical practice with frequent visits, antibiotics (per oral or parenteral) or operative intervention if severity warrants it.

6.9 Statistics

6.9.1 A description of the statistical methods to be employed, including timing of any planned interim analysis(ses).

Statistical analysis

Statistical tests will be performed and reported according to the intention-to-treat principle. All patients randomized and 1st dose taken will be included in the statistical evaluation. Data will be presented as mean and standard deviation or median and range, as appropriate, for continuous variables and as numbers and proportions for categorical variables.

The primary analysis of the CTS symptom severity score at 10 weeks will be performed using a mixed model analysis of repeated measures (baseline, 5 weeks, 10 weeks) with treatment (placebo or Depomedrol) as random factor and time as fixed factor in a 1st step and with the interaction term of treatment and time in 2nd step. The results will be presented as differences in mean score change over time (baseline to 10 weeks) and 95% confidence intervals. The secondary analysis of the symptom severity score will be done using mixed model with treatment (placebo or Depomedrol) as a random factor and time (baseline, 5 weeks, 10 weeks, 24 weeks, 52 weeks) as fixed factor and surgery as covariate; 1st step without interaction terms and 2nd step with two-way interaction of treatment and time. The results will be presented as differences in mean score change over time and 95% confidence intervals.

The primary analysis of rate of surgery will be done using Fishers exact test (univariate) and logistic regression (multivariate) with adjustment for baseline differences. The issue of multiplicity is addressed by performing the analysis in the following priority; 52 weeks, 24 weeks, and 12 weeks. The results will be presented as differences in rate of surgery between the groups (Depomedrol vs placebo) and adjusted odds ratio (95% confidence interval) of having surgery at each time point.

The expected dose-response reaction with higher effect of high dose will be explored by prioritizing group comparison according to 6.7.1

The effect sizes for symptom severity and *Quick*DASH will be compared in the two groups using 95% confidence intervals calculated using jackknife procedures (effect size of 0.2 to 0.4 is considered small, 0.5 to 0.8 moderate and greater than 0.8 as large effect size).[18]

The Adverse Events will be presented in tables, and statistically analyzed when appropriate.
Interim analysis will be performed by fellow researcher not involved in study after completion of half of the patients if the adverse events are not in accordance to the product resumes.

6.9.2 The number of subjects planned to be enrolled. In multicentre trials, the numbers of enrolled subjects projected for each trial site should be specified. Reason for choice of sample size, including reflections on (or calculations of) the power of the trial and clinical justification.
<u>Number of patients</u>
Based on previous studies patients with idiopathic CTS improve by about 1.3 points on the CTS symptom severity scale at 3 weeks after surgery and by 0.8 points (SD 0.8) at 2 weeks after steroid injection. With 5% significance level, and two-sided statistical tests, randomization of 90 patients will provide 90% statistical power to detect a true difference of at least 0.8 point between an intervention group and the placebo group (assumed SD 0.9) at 10 weeks after treatment and reduction in rate of surgery from assumed 80% in placebo to 40% in intervention group at 52 weeks after treatment. An improvement of less than 0.8 point on the CTS symptoms severity scale in this population cannot be considered as adequate improvement.

6.9.3 The level of significance to be used.
5%
6.9.4 Criteria for the termination of the trial.
Termination of the study will be carried out in case the SAE frequency of the interim analysis is considerably higher than in the drugs resumes. The statistician will be involved in calculating correlated frequency since the population is too small to make a proper estimate of rare SAEs

6.9.5 Procedure for accounting for missing, unused, and spurious data.
Data quality will be checked. Efforts will be done to avoid missing data and achieve completeness (including contacting patients by telephone to obtain missing data when appropriate). Finally, the mixed model analysis accounts for missing data. For the rate of surgery only patients with data at 5 and 10 weeks can be included in analysis.
6.9.6 Procedures for reporting any deviation(s) from the original statistical plan (any deviation(s) from the original statistical plan should be described and justified in protocol and/or in the final report, as appropriate).
<u>Internal report</u>
A statistical evaluation will be performed within three months of trial termination. On the basis of this statistical report an internal integrated report will be prepared by the principal investigator.
If the trial is terminated prematurely for any reason an abbreviated report will be prepared.

6.9.7 The selection of subjects to be included in the analyses (e.g. all randomized subjects, all dosed subjects, all eligible subjects, evaluable subjects).
All randomized subjects who receive a dose.
© EMEA 2006 39
6.10 Direct Access to Source Data/Documents
The sponsor should ensure that it is specified in the protocol or other written agreement that

the investigator(s)/institution(s) will permit trial-related monitoring, audits, IRB/IEC review, and regulatory inspection(s), providing direct access to source data/documents.

6.11 Quality Control and Quality Assurance

The trial will be monitored by health professionals with experience of clinical trials and documented knowledge of GCP. The monitor will have access to and patient files, CRF and questionnaires. Monitoring will be carried out in accordance to ICH-GCP

6.12 Ethics and Health Authorities

Description of ethical considerations relating to the trial and the health authorities application.

Ethics committee approval

The study protocol has been submitted to the Ethics Committee at the Faculty of Medicine, Lund University for approval and an application will be submitted for approval to ´the Medical Products Agency in Uppsala before the performance of the clinical trial.

6.13 Data Handling and Record Keeping

Data management

When CRFs and outcome questionnaires have been verified, they will be scanned into a computer database for further data handling and statistical evaluation.

Prior to breaking of the code all decisions on the evaluability of each individual patient in the statistical analyses will be made and documented under the process of Clean File.

Record Keeping

The Patient related papers will be kept in a locked area in the investigators office in the outpatients department. The patient files are in electronic form in the health care system for hospital records.

6.14 Financing and Insurance

Financing and insurance if not addressed in a separate agreement.

The study will be conducted within the usual health care and the costs related to additional follow-up evaluations will be financed with research funds.

All patients within a Clinical trial are protected by the general patient insurance and the insurance for medical treatment will be used in case of injection related complication.

6.15 Publication Policy

Publication policy, if not addressed in a separate agreement.

Publication

The performing of this study and the result of it will be described in manuscripts that will be presented to those granting financial support and submitted for publication in scientific medical journal.

6.16 Supplements

(NOTE: Since the protocol and the clinical trial/study report are closely related, further relevant information can be found in the ICH Guideline for Structure and Content of Clinical

2. Protocol for the Extended Follow-up

Extended follow-up of a randomized controlled trial of local steroid injection in carpal tunnel syndrome

Isam Atroshi, MD, PhD,[1] Manfred Hofer, BSc[2]

[1]Professor of Orthopedics, Department of Clinical Sciences Lund, Lund University, Lund and Department of Orthopedics Hässleholm-Kristianstad, Hässleholm Hospital, Hässleholm, Sweden.

[2]Physiotherapist, Department of Orthopedics Hässleholm-Kristianstad, Hässleholm Hospital, Hässleholm, Sweden.

Principal investigator:

Isam Atroshi, Department of Orthopedics, Kristianstad Hospital, SE 29185 Kristianstad, Sweden.

Tel: +46443091279

E-mail: Isam.Atroshi@med.lu.se

Background

Local steroid injection is a common non-operative treatment in patients with carpal tunnel syndrome (CTS). According to a survey of US hand surgeons, 81% used steroid injection before considering surgery.[1] A systematic review of studies published through May 2006 found only two good-quality double-blind placebo-controlled trials and concluded that local corticosteroid injection provides clinical improvement one month after injection compared to placebo but none of the randomized studies remained blinded beyond one month.[2] One of the studies, involving 81 patients, reported significantly better patient satisfaction 2 weeks after 6 mg betamethasone.[3] The second study, involving 60 patients, reported better symptomatic improvement 1 month after 40 mg methylprednisolone.[4] In a randomized study published after that systematic review's search interval, 35 patients received 10 mg triamcenolone (24 received two injections with 1-week interval) and 31 patients received saline, but blinding was terminated 3 weeks after injection at which time the triamcenolone group had significantly larger CTS symptom severity score improvement than the saline group (mean difference 0.64 on a 1 to 5 scale).[5]

Between November 2008 and March 2012 we performed a single-center randomized double-blind placebo-controlled trial to assess the efficacy of local injection of 2 different doses of methylprednisolone (80 mg and 40 mg) in patients with CTS, aged 18 to 70 years, not previously treated with steroid injection.[6] The primary endpoints were change in the CTS symptom severity score at 10 weeks and rate of carpal tunnel release surgery on the study hand at 1 year. We

randomized 111 patients (37 in each of the 3 groups: 80 mg methylprednisolone, 40 mg methylprednisolone and placebo). We found that Improvement in the symptom severity score at 10 weeks was significantly greater in patients who received 80 mg or 40 mg methylprednisolone than placebo (difference in change from baseline -0.64 [CI, -1.06 to -0.21] and 0.88 [CI, -1.30 to -0.46], respectively, $P<0.01$) but there were no significant differences in the scores at 1 year. During 1 year after injection, carpal tunnel release surgery on the study hand was done in 73% (27 patients) in the 80-mg group, 81% (30 patients) in the 40-mg group, and 92% (34 patients) in the placebo group. Patients who received 80 mg methylprednisolone were significantly less likely than those who received placebo to undergo surgery during 1 year (odds ratio 0.24; 95% CI, 0.06-0.95). To our knowledge this trial is still the only randomized trial with a 1-year follow-up during which blinding of patients and investigators was maintained until all patients had completed the final follow-up.

No randomized studies have assessed outcomes of steroid injection in CTS beyond 1 year. A common argument against routine use of steroid injections for CTS is the lack of evidence regarding durability of the benefit. In contrast, symptom relief after surgery is durable for more than 12 years.[7] Another question that has been recently discussed is whether steroid injection may worsen the outcomes of subsequent carpal tunnel release;.[8] this has not been previously investigated in prospective study.

We plan to conduct an extended follow-up of our randomized controlled trial 5 to 6 years after injection. The objective is to determine (1) whether the beneficial effect of local injection of 80 mg methylprednisolone compared to placebo, as measured with the proportion of patients who had required surgery within 1 year after injection, persists up to 5 years, (2) whether the magnitude of symptom improvement is larger in patients who had surgery after injection than in patients who did not have surgery after steroid injection, and (3) whether the magnitude of improvement in symptom severity differs in patients who had surgery after steroid injection and those who had surgery after placebo injection.

Patients and Methods

Trial's eligibility criteria, randomization and assessments

The trial has been described previously.[6;9] In a double-blind placebo-controlled trial patients who met the trial's eligibility criteria were randomized (computer-generated randomization list; 1:1:1 ratio) to 1 of 3 groups; 80 mg methylprednisolone, 40 mg methylprednisolone and placebo. The inclusion criteria were primary idiopathic CTS, age 18-70 years, symptoms of classic or probable CTS (numbness and/or tingling in at least 2 of the 4 median nerve innervated fingers) according to the Katz diagnostic criteria [10], failed treatment with wrist splinting (2 months), and nerve conduction tests showing median neuropathy at the wrist or, if normal, two orthopedic surgeons independently diagnose the patient with CTS and symptom severity that warranted referral for consideration for surgery. The exclusion criteria were previous steroid injection, thenar muscle atrophy, sensory loss (two-point discrimination >8 mm), diabetes, thyroid disorder, inflammatory disease, vibration-induced neuropathy, polyneuropathy, current pregnancy, previous carpal tunnel release, surgery in the contralateral hand in the past 2 months, inability to respond to questionnaires, severe medical illness, and drugs or alcohol abuse. The primary outcomes were the improvement in symptom severity score at 10 weeks and the rate of surgery at 1 year.

At baseline the patients completed the CTS symptom severity scale, the 11-item disabilities of the arm, shoulder and hand (DASH) scale and a 2-item pain scale. The 11-item CTS symptom severity scale measures severity, frequency and duration of night and daytime pain and

numbness/tingling [11]; score from 1 (no symptoms) to 5 (most severe). The 11-item DASH measures difficulties in performing daily activities [12]; score from 0 (no disability) to 100 (worst). The 2-item bodily pain scale yields a score from 0 (worst) to 100.

Physical examination performed by the study physical therapist included measurement of hand strength (grip and pinch) and sensation (monofilament and two-point discrimination). Nerve conduction testing was performed, measuring median nerve wrist-to-index finger sensory latency and wrist-to-ring finger median-ulnar sensory latency difference, and classified by a neurophysiologist as severe, moderate, mild or normal. Absent response or sensory latency difference ≥1.7 milliseconds (ms) were considered as severe, 1.0-1.6 ms as moderate, 0.6-0.9 ms as mild, and below 0.6 ms as normal.

Of the 111 patients randomized, 37 patients were assigned to 80 mg methylprednisolone, 37 patients to 40 mg methylprednisolone, and 37 patients to placebo (normal saline), each combined with 1 ml lidocaine for a total injected volume of 3 ml. All patients received the intervention to which they were assigned. Follow-up evaluations were done at 5 weeks, 10 weeks, 6 months and 1 year. At each follow-up the patients completed the same scales as in the baseline assessment and an additional visual analog scale (VAS) about treatment satisfaction, with range from 0 (very dissatisfied) to 100 (completely satisfied). At the 1-year follow-up a physical examination similar to that done at baseline was also performed. No repeat injections

were given during the trial. Patients who did not improve or experienced recurrent symptom after injection were offered carpal tunnel release surgery.

Extended follow-up protocol

All 111 trial participants will be contacted by telephone by a researcher and given information about this extended follow-up. A questionnaire together with written information and consent forms will be sent by mail. The questionnaire consists of the CTS symptom severity sale, 11-item DASH scale, bodily pain scale, and a treatment satisfaction VAS, that were used in previous follow-up evaluations. The questionnaire will also include the 6-item CTS symptoms scale (CTS-6) and the palmar pain scale. The CTS-6 is a 6-item scale that measures numbness, tingling and pain in the hand (score range 1 to 5).[13] The palmar pain scale is a 2-item scale that measures pain in the proximal palm and related activity limitations (score range 0 to 100).[14] The patients will also be asked about whether they had undergone carpal tunnel release surgery on the study hand after they received the injection within the trial. Patients' records will be reviewed to verify data about subsequent surgery on the study hand.

Primary outcomes

The primary outcomes are (1) the difference between the 80-mg methylprednisolone group and the placebo group in the proportion of patients who have had carpal tunnel release surgery on the study hand at 5 years after injection, and (2) the difference in change in symptom severity score from baseline to 5 to 6 years between patients in the 80-mg methylprednisolone group who had not undergone carpal tunnel release surgery on the study hand and patients in the 80-mg or 40-mg methylprednisolone groups who had undergone surgery after injection.

Secondary outcomes

The secondary outcomes are (1) palmar pain scores at 5 to 6 years, (2) change in 11-item DASH and bodily pain scores from baseline and from 1 year to 5 to 6 years, (3) change in satisfaction scores from 1 year to 5 to 6 years.

Sample size

Of the 37 patients randomized to each of the 3 groups the number of patients who have had surgery within 1 year after injection was 27 in the 80-mg methylprednisolone group and 34 in the placebo group. Assuming a complete follow-up can be achieved the sample size would be adequate to detect a statistically significant difference in rate of surgery at 5 years (primary outcome), using 2-sided test, p value of 0.05 and 80% power.

Statistical Analysis

The Chi-square test will be used to compare the 80-mg methylprednisolone group and the placebo group with regard to the proportion of patients who have had carpal tunnel release surgery on the study hand within 5 years after injection (primary outcome). The change in symptom severity score in patients in the 80-mg methylprednisolone group who did not have surgery on the study hand after injection will be compared with the change in symptom severity score in patients in any group who had surgery on the study hand after injection (co-primary outcome) using analysis of covariance (ANCOVA) adjusting for sex, age, dominance of the study hand and baseline symptom severity score. A similar comparison will be made for the 11-item DASH and the bodily pain scores. The palmar pain score at 5 to 6 years will be compared

between the groups using the independent-samples t-test and also ANCOVA to adjust for sex, age and dominance of the study hand. Patients who had surgery after methylprednisolone injection will be compared with patients who had surgery after placebo injection with regard to change from baseline to 5 to 6 years in the symptom severity score, 11-item DASH scale score, bodily pain score and satisfaction score using ANCOVA adjusting for sex, age, dominance of the study hand and respective baseline score. To identify potential predictive factors for long-term benefit after 80 mg methylprednisolone injection, surgery within 5 years will be analyzed according to the variables sex, age, dominance of the study hand, baseline symptom severity score (≥3 versus < 3) and baseline median nerve conduction abnormality (severe/moderate vs mild/normal).

All statistical tests will be 2-sided and a p value of less than 0.05 will indicate statistical significance.

Discussion

This extended follow-up of our randomized double-blind placebo-controlled trial will provide new evidence about the durability of the effect of local steroid injection in the treatment of CTS. The trial has previously shown that a significantly larger proportion of patients who received 80 mg methylprednisolone did not need surgery as compared to patients who received placebo. If this benefit persists up to 5 years it would be an important advantage supporting use of local steroid injection before considering surgery. Choosing not to have surgery does not necessarily indicate that the patients no longer have symptoms. Therefore, an important question that the extended follow-up will address is whether or not patients who have not undergone surgery have a higher symptoms level than patients who have had surgery. One limitation of this extended follow-up is that the trial was blinded only up to the time when all patients had completed the 1-year follow-up, after which patients were informed of the type of injection they had received, in accordance with the trial protocol. However, the importance of blinding is probably greater early after the injection than beyond 1 year.

References

1. Leinberry CF, Rivlin M, Maltenfort M et al. Treatment of carpal tunnel syndrome by members of the American Society for Surgery of the Hand: a 25-year perspective. *J Hand Surg Am*. 2012;37:1997-2003.

2. Marshall S, Tardif G, Ashworth N. Local corticosteroid injection for carpal tunnel syndrome. *Cochrane Database Syst Rev*. 2007;CD001554.

3. Armstrong T, Devor W, Borschel L, Contreras R. Intracarpal steroid injection is safe and effective for short-term management of carpal tunnel syndrome. *Muscle Nerve*. 2004;29:82-88.

4. Dammers JW, Veering MM, Vermeulen M. Injection with methylprednisolone proximal to the carpal tunnel: randomised double blind trial. *BMJ*. 1999;319:884-886.

5. Peters-Veluthamaningal C, Winters JC, Groenier KH, Meyboom-de Jong B. Randomised controlled trial of local corticosteroid injections for carpal tunnel syndrome in general practice. *BMC Fam Pract*. 2010;11:54.

6. Atroshi I, Flondell M, Hofer M, Ranstam J. Methylprednisolone injections for the carpal tunnel syndrome: a randomized, placebo-controlled trial. *Ann Intern Med*. 2013;159:309-317.

7. Atroshi I, Hofer M, Larsson GU, Ranstam J. Extended Follow-up of a Randomized Clinical Trial of Open vs Endoscopic Release Surgery for Carpal Tunnel Syndrome. *JAMA*. 2015;314:1399-1401.

8. Bland JD, Ashworth NL. Does prior local corticosteroid injection prejudice the outcome of subsequent carpal tunnel decompression? *J Hand Surg Eur Vol*. 2015.

9. Flondell M, Hofer M, Bjork J, Atroshi I. Local steroid injection for moderately severe idiopathic carpal tunnel syndrome: protocol of a randomized double-blind placebo-controlled trial (NCT 00806871). *BMC Musculoskelet Disord*. 2010;11:76.

10. Katz JN, Stirrat CR. A self-administered hand diagram for the diagnosis of carpal tunnel syndrome. *J Hand Surg*. 1990;15A:360-363.

11. Levine DW, Simmons BP, Koris MJ et al. A self-administered questionnaire for the assessment of severity of symptoms and functional status in carpal tunnel syndrome. *Journal of Bone and Joint Surgery [Am]*. 1993;75:1585-1592.

12. Beaton DE, Wright JG, Katz JN. Development of the QuickDASH: comparison of three item-reduction approaches. *J Bone Joint Surg Am*. 2005;87:1038-1046.

13. Atroshi I, Lyren PE, Gummesson C. The 6-item CTS symptoms scale: a brief outcomes measure for carpal tunnel syndrome. *Qual Life Res*. 2009;18:347-358.

14. Atroshi I, Lyren PE, Ornstein E, Gummesson C. The six-item CTS symptoms scale and palmar pain scale in carpal tunnel syndrome. *J Hand Surg Am*. 2011;36:788-794.

3. Ethical Review Attachments for the Extended Follow-up

9. Förteckning över bilagor (Se p. 9 i Vägledning till ansökan)

Dokument som, i tillämpliga fall, ska bifogas *om inte motsvarande information finns i blanketten* har markerats med x. Markera de bilagor som skickas in med denna ansökan.

Insänd med ansökan	Bil nr	Beskrivning	Klinisk läkemedels-prövning	Annan forskning
☐	1	Deltagande forskningshuvudmän och medverkande forskare (kontaktpersoner) vid forskning där mer än en forskningshuvudman deltar. Se p. 1:4	x	x
☒	2	För fackmän avsedd forskningsplan, vid behov även för lekmän avsedd bilaga. Se p. 2:1 och Vägledning till forskningsplan/forskningsprotokoll (program)	x	x
☐	3	Annonsmaterial för rekrytering av forskningspersoner. Se p. 3:1 och i Vägledning till ansökan p. 3:1	x	x
☒	4	Skriftlig information till dem som tillfrågas. Se p. 4:1 och Vägledning till forskningspersonsinformation och (i förekommande fall) separat samtyckesformulär	x	x
☒	5	Enkät, frågeformulär. Se p. 2:4	x	x
☐	6	Gemensam EU blankett (gäller fr.o.m. den 1 maj 2004), gäller även vid ändring. För information se Läkemedelsverkets hemsida, www.lakemedelsverket.se	x	
☐	7	Sammanfattning av protokollet på svenska	x	
☐	8	Prövarhandbok alt. bipacksedel/produktresumé/IB	x	
☒	9	Intyg från verksamhetschef/motsv. om resurser för forskningspersonernas säkerhet. Se p. 1:5 och förslag till utformning av resursintyg i Vägledning till ansökan p. 1:5	x	x
☒	10	CV för forskare (samma som p. 1:3) med huvudansvar för genomförandet, redovisa forskarens (- arnas) kompetens av relevans för studien. Se Vägledning till ansökan p. 1:3	x	x
☐	11	Beskrivning av ersättning till forskningspersoner. Se p. 3:6 och i Vägledning till ansökan p. 3:6	x	x

Övriga bilagor som bifogas ansökan:

Bilaga 1: Etikbeslut för den ursprungliga studien

Bilaga 3: Atroshi I, Flondell M, Hofer M, Ranstam J. Methylprednisolone injections for the carpal tunnel syndrome: a randomized, placebo-controlled trial. Ann Intern Med 2013;159:309-317.

REGIONALA ETIKPRÖV-NINGSNÄMNDEN I LUND, Avd 1
Box 133
221 00 LUND
Jacob Branting, adm sekreterare
046-222 41 80

PROTOKOLL 2008/8
Sammanträde 2008-09-04 kl. 13.00-16.00
Medicinska fakultetens sammanträdesrum
Stora Algatan 4, Lund

ANKOM
ORT KLIN

NÄRVARANDE

Ledamöter

Ordförande
Gunilla Hedesten Nordin

Ledamöter med vetenskaplig kompetens
Jan Rosenquist, vetenskaplig sekreterare
Elisabet Englund
Lars Edvinsson
Marita Hilliges
Eva Maria Fenyö deltog ej i 436/08 pga jäv
Holger Luthman, deltog ej i 430/08 pga jäv
Ingalill Rahm Hallberg, deltog ej i 425 pga jäv
Margareta Östman

Företrädare för allmänna intressen
Karolina Algotsson
Patrik Åkesson deltog ej i 366/08, 381/08 och 401/08
Lars Olin
Inger Sandell Norén
Gunnel Wallin deltog ej i 366/08, 381/08 och 401

Övriga närvarande

Administrativ sekreterare
Jacob Branting

-------------------------------**utdrag**---

§ 3 **Ansökningar som varit upptagna på föredragningslista tillhörande tidigare möten**

Punkten 3.1

Dnr 119/2008
Föredragande
Margareta Östman

Forskningshuvudman
Region Skåne

Forskare som genomför projektet (kontaktperson)
Magnus Flondell

Projekttitel
Utvärdering av lokal injektion av kortison vid behandling av karpaltunnelsyndrom.

Beslut
Ansökan godkänns

Att protokollsutdraget överensstämmer med originalet intygar:

Jacob Branting, administrativ sekreterare

Exp till:
Magnus Flondell
Peter Kalén

2016-02-01

Hej!

För drygt 5 år sedan deltog Du i en studie på Ortopediska kliniken Hässleholm-Kristianstad om nyttan av lokal kortisoninjektion för behandling av nervinklämning i handleden (karpaltunnelsyndrom). Vi tackar dig igen för ditt deltagande som har bidragit till viktig kunskap om behandling av karpaltunnelsyndrom som är en vanlig orsak till handbesvär.

Vi skulle nu vilja göra en ny uppföljning med enkät för att undersöka hur resultaten är 5 år efter behandlingen. Vi vill fråga dig om Du skulle vilja delta i en enkätuppföljning liknande den Du besvarade 1 år efter Du fick studiebehandlingen.

Syftet med denna 5-års uppföljning är att få mer kunskap om effekten av lokal kortisoninjektion vid karpaltunnelsyndrom på längre sikt. Informationen från studien kommer att hjälpa oss när vi diskuterar med patienter som har karpaltunnelsyndrom val av lämplig behandling samt för- och nackdelar och prognos för olika behandlingsmetoder.

Deltagandet i denna enkätuppföljning är helt frivilligt. Svaren kommer att bearbetas helt konfidentiellt och resultaten presenteras på gruppnivå. Ingen deltagare kommer att kunna identifieras.

Om Du är villig att delta i denna enkätuppföljning så skulle vi vara mycket tacksamma om du skriver under ditt samtycke till deltagandet samt fyller i den bifogade enkäten och returnera dem i det bifogade svarskuvertet.

Om Du har några frågor är Du välkommen att kontakta sjuksköterska Ingela Ranebo på Hässleholms sjukhus (0451 - 29 88 36) eller sjuksköterska Pia Gunnarsson på ortopedmottagningen i Kristianstad (044 – 309 12 60).

Tack för Din medverkan.

Med vänlig hälsning

Ansvarig för studien är:

Isam Atroshi,
Professor, överläkare
Ortopedmottagningen, Centralsjukhuset i Kristianstad

Undertecknad har fått skriftlig information om enkätstudien som syftar att undersöka 5-års resultat efter lokal kortisoninjektion för karpaltunnelsyndrom.

Jag samtycker till att delta. Jag är också informerad om att deltagandet är frivilligt.

Datum:

Namn: ____

Svara på följande frågor med avseende på ○ Höger ○ Vänster hand.

Frågorna avser Dina symtom en vanlig 24 timmars period under de **senaste två veckorna**.
(Fyll ringen vid ett svarsalternativ för varje fråga)

Hur mycket värk eller smärta har Du i hand eller handled nattetid?

- ○ Jag har inte någon värk eller smärta i hand eller handled nattetid
- ○ Lätt värk eller smärta
- ○ Måttlig
- ○ Svår
- ○ Mycket svår

Hur ofta, i genomsnitt, vaknade Du av att Du hade värk eller smärta i handen eller handleden under en natt de senaste två veckorna?

- ○ Aldrig
- ○ En gång
- ○ Två eller tre gånger
- ○ Fyra eller fem gånger
- ○ Mer än fem gånger

Har Du vanligtvis värk eller smärta i handen eller handleden dagtid?

- ○ Jag har inte någon värk eller smärta i hand eller handled dagtid
- ○ Lätt värk eller smärta
- ○ Måttlig
- ○ Svår
- ○ Mycket svår

Hur ofta, i genomsnitt, har Du värk eller smärta i handen eller handleden, dagtid?

- ○ Aldrig
- ○ En gång
- ○ Två eller tre gånger
- ○ Fyra eller fem gånger
- ○ Mer än fem gånger

Hur länge, i genomsnitt, varar varje stund Du har värk eller smärta i handen eller handleden dagtid?

- ○ Jag har aldrig värk eller smärta i hand eller handled dagtid
- ○ 10 minuter eller mindre
- ○ Mer än 10 minuter, upptill en timma
- ○ Mer än en timma
- ○ Konstant hela dagen

Har Du domningar i handen?

- O Nej
- O Lätta domningar
- O Måttliga domningar
- O Svåra domningar
- O Mycket svåra domningar

Är Du svag i handen eller handleden?

- O Nej
- O Lätt svaghet
- O Måttligt
- O Mycket
- O Väldigt mycket

Känner Du stickningar i handen?

- O Nej
- O Lätta stickningar
- O Måttliga
- O Svåra
- O Mycket svåra

Hur svåra är domningarna eller stickningarna nattetid?

- O Jag har inga domningar eller stickningar nattetid
- O Lätta
- O Måttliga
- O Svåra
- O Mycket svåra

Hur ofta, i genomsnitt, vaknade Du av stickningar eller domningar i handen under en natt den senaste veckan**?**

- O Aldrig
- O En gång
- O Två eller tre gånger
- O Fyra eller fem gånger
- O Mer än fem gånger

Har Du svårt att gripa om och använda små föremål såsom nycklar och pennor?

- O Ingen svårighet
- O Viss svårighet
- O Måttlig svårighet
- O Stor svårighet
- O Mycket stor svårighet

4934115355

Denna enkät berör Dina symtom och Din förmåga att utföra vissa aktiviteter.
Svara på varje fråga, baserat på hur Du har mått den senaste veckan. Om det är någon aktivitet Du inte har utfört den senaste veckan får Du kryssa för det svar som Du bedömer stämmer bäst om Du hade utfört aktiviteten.

Gradera Din förmåga att utföra följande aktiviteter under den senaste veckan genom att kryssa för ett svarsalternativ för varje fråga

	Ingen svårighet	Viss svårighet	Måttlig svårighet	Stor svårighet	Omöjligt att göra
1. Öppna en ny burk eller hårt sittande lock	☐	☐	☐	☐	☐
2. Utföra tunga hushållssysslor (t ex tvätta golv, putsa fönster, hänga tvätt)	☐	☐	☐	☐	☐
3. Bära matkassar eller portfölj	☐	☐	☐	☐	☐
4. Tvätta Din rygg	☐	☐	☐	☐	☐
5. Använda en kniv för att skära upp maten	☐	☐	☐	☐	☐
6. Fritidsaktiviteter som tar upp viss kraft eller stöt genom arm, axel eller hand (t ex spela golf, använda hammare, spela tennis, skytte, bowling)	☐	☐	☐	☐	☐

7. Under den senaste veckan, i vilken utsträckning har Dina arm-, axel- eller handproblem stört Ditt vanliga umgänge med anhöriga, vänner, grannar eller andra?

☐ Inte alls ☐ Lite ☐ Måttligt ☐ Mycket ☐ Väldigt mycket

8. Under den senaste veckan, i vilken utsträckning har Dina arm-, axel- eller handproblem stört Ditt vanliga arbete eller andra dagliga aktiviteter?

☐ Inte alls ☐ Lite ☐ Måttligt ☐ Mycket ☐ Väldigt mycket

Ange svårighetsgraden på Dina symtom den senaste veckan:

	Ingen	Lätt	Måttlig	Svår	Mycket svår
9. Värk/smärta i arm, axel eller hand	☐	☐	☐	☐	☐
10. Stickningar (sockerdrickskänsla) i arm, axel eller hand	☐	☐	☐	☐	☐

11. Har Du haft svårt att sova, under den senaste veckan, på grund av värk/smärta i arm, axel eller hand?

☐ Inte alls ☐ Viss svårighet ☐ Måttlig svårighet ☐ Stor svårighet ☐ Mycket stor svårighet

QuickDASH Gummesson/Atroshi 2006

7404115350

Svara på följande frågor med avseende på ○ Höger ○ Vänster hand

Frågorna avser Dina handsymtom en vanlig 24 timmars period under de senaste två veckorna.
(Kryssa för ett svarsalternativ för varje fråga)

Hur svåra är följande symptom i Din hand?	Ingen	Lätt	Måttlig	Svår	Mycket svår
1. Värk nattetid	☐	☐	☐	☐	☐
2. Värk dagtid	☐	☐	☐	☐	☐
3. Domningar eller stickningar nattetid	☐	☐	☐	☐	☐
4. Domningar eller stickningar dagtid	☐	☐	☐	☐	☐

Hur ofta vaknar Du på natten på grund av följande symptom i Din hand?	Aldrig	En gång	2 eller 3 gånger	4 eller 5 gånger	mer än 5 gånger
5. Värk	☐	☐	☐	☐	☐
6. Domningar eller stickningar nattetid	☐	☐	☐	☐	☐

Hur mycket värk, smärta eller ömhet har Du vid insidan av handleden (mellan handleden och handflatan)?

○ Ingen
○ mycket lätt
○ Lätt
○ Måttlig
○ Svår
○ mycket svår

Hur mycket har värk, smärta eller ömhet vid insidan av handleden hindrat Dig i aktiviteter?

○ inte alls
○ lite
○ måttligt
○ mycket
○ väldigt mycket

Följande frågor gäller om Du har svarat att Du haft domningar eller stickningar i din _____ hand senaste veckorna.

Hur ofta känner Du domningar eller stickningar i din _____ hand?

- ☐ Dagligen
- ☐ Några gånger i veckan
- ☐ Några gånger i månaden
- ☐ Någon gång i månaden

Var i din _____ hand känner du domningarna eller stickningarna (Du får kryssa på flera svarsalternativ)?

- ☐ Framsidan av fingrarna (samma sida som handflatan)
- ☐ Baksidan av fingrarna (handryggsidan)
- ☐ Båda
- ☐ Inte i fingrarna

I vilket finger eller fingrar i din ______ hand känner Du domningarna eller stickningarna (Du får kryssa på flera svarsalternativ)?

- ☐ Tumme
- ☐ Pekfinger
- ☐ Långfinger
- ☐ Ringfinger
- ☐ Lillfinger
- ☐ Inte i fingrarna

Sätt ett kryss på det streck som Du tycker motsvarar hur nöjd Du är med behandlingsresultatet i din _____ hand

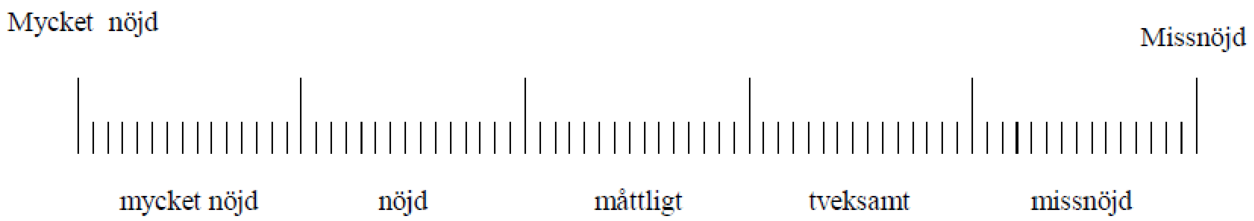

Har Du blivit opererad i dina händer för nervinklämning (karpaltunnelsyndrom)?

- ☐ Ja, höger hand
- ☐ Ja, vänster hand
- ☐ Nej
