# Supplementary material for: Extended Follow-up of Local Steroid Injection for Carpal Tunnel Syndrome: A Randomized Clinical Trial
Source: JAMA Netw Open. 2021 Oct 22;4(10):e2130753. doi: 10.1001/jamanetworkopen.2021.30753 (PMC8536954; doi:10.1001/jamanetworkopen.2021.30753)
Supplement: Supplement 2. — eAppendix. Trial Eligibility Criteria eFigure. Trial Flowchart [file jamanetwopen-e2130753-s002.pdf]

Supplemental Online Content

Hofer M, Ranstam J, Atroshi I. Extended follow-up of local steroid injection for carpal tunnel syndrome: a randomized clinical trial. *JAMA Netw Open*. 2021;4(10):e2130753. doi:10.1001/jamanetworkopen.2021.30753

eAppendix. Trial Eligibility Criteria

eFigure. Trial Flowchart

This supplemental material has been provided by the authors to give readers additional information about their work.

© 2021 Hofer M et al. *JAMA Network Open.*

eAppendix. Trial Eligibility Criteria

Inclusion criteria

• Primary idiopathic CTS

• Patient age 18-70 years

• Symptoms of classic or probable CTS (numbness and/or tingling in at least 2 of the 4 median nerve innervated fingers) according to the Katz diagnostic criteria

• Failed 2-month treatment with wrist splinting

• Nerve conduction tests showing median neuropathy at the wrist or, if normal, two orthopedic surgeons independently diagnose the patient with CTS

• Symptom severity that warranted referral for consideration for surgery

Exclusion criteria

• Previous steroid injection

• Thenar muscle atrophy

• Sensory loss (two-point discrimination >8 mm)

• Medical conditions: diabetes, thyroid disorder, inflammatory disease

• Vibration-induced neuropathy or polyneuropathy

• Current pregnancy

• Previous carpal tunnel release surgery in the contralateral hand in the past 2 months

• Inability to respond to questionnaires

• Severe medical illness

• Known abuse of drugs or alcohol

© 2021 Hofer M et al. *JAMA Network Open.*

eFigure. Trial Flowchart

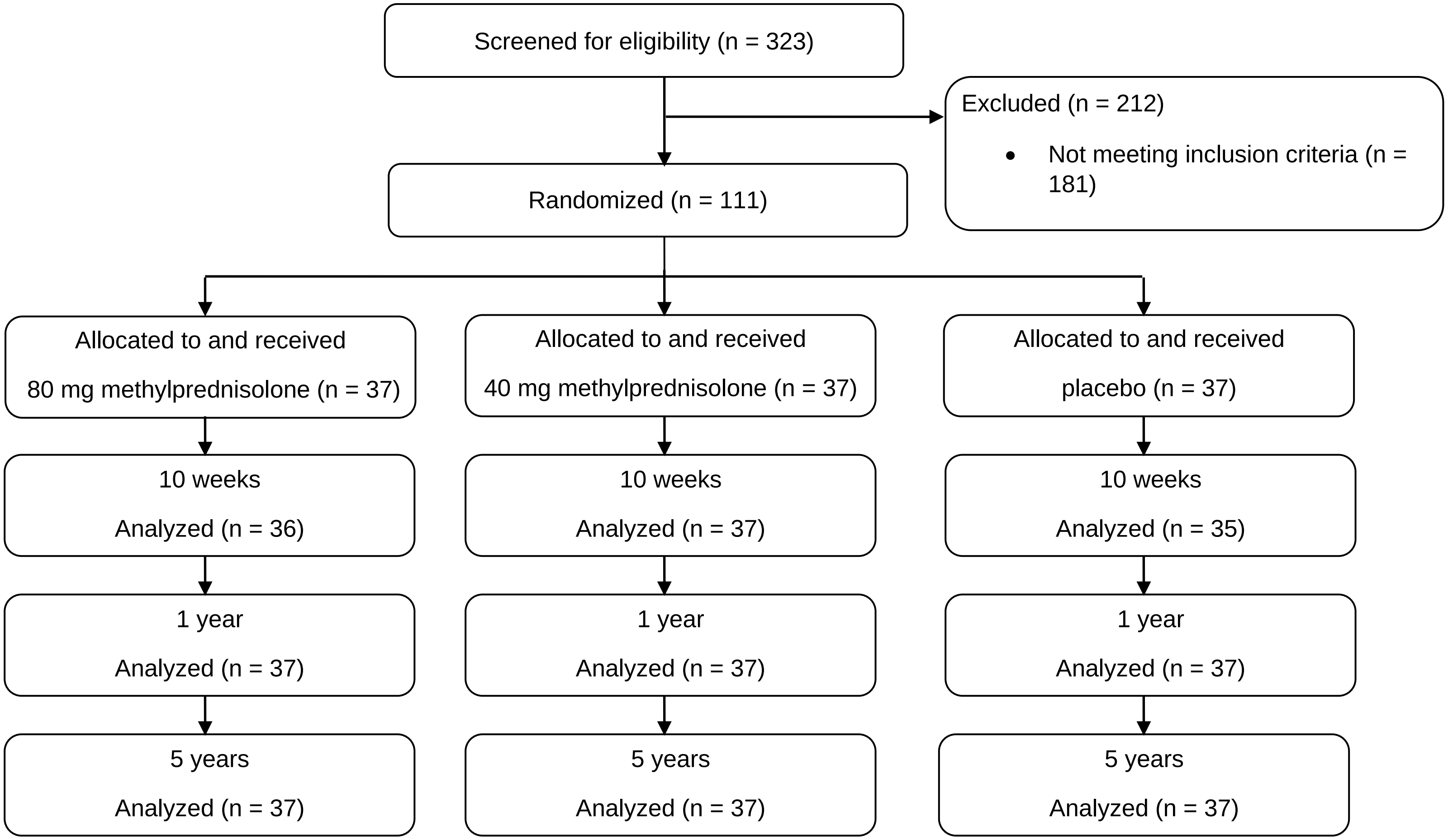

© 2021 Hofer M et al. *JAMA Network Open.*
