# Supplementary material for: Extended Follow-up of Local Steroid Injection for Carpal Tunnel Syndrome: A Randomized Clinical Trial
Source: JAMA Netw Open. 2021 Oct 22;4(10):e2130753. doi: 10.1001/jamanetworkopen.2021.30753 (PMC8536954; doi:10.1001/jamanetworkopen.2021.30753)
Supplement: Supplement 3. — Data Sharing Statement [file jamanetwopen-e2130753-s003.pdf]

Data Sharing Statement

Hofer. Extended Follow-up of Local Steroid Injection for Carpal Tunnel Syndrome. *JAMA Netw Open*. Published October 22, 2021. doi:10.1001/jamanetworkopen.2021.30753

Data
Data available: No

Additional Information
Explanation for why data not available: Making individual patient data publicly available will be outside the Ethical Review Board permission. Individual patient data will be provided to researchers on reasonable request to the correspodnig author.
